# Isolated Left Innominate Artery with a Right Arch and Bilateral Patent Arterial Ducts in a Patient with 22q11 Microdeletion: Report of a Rare Case

**DOI:** 10.34172/jcvtr.33194

**Published:** 2026-06-30

**Authors:** Haytham Bou Hussein, Rim Salameh, Rana Zareef, Issam El Rassi, Fadi Bitar, Ziad Bulbul

**Affiliations:** ^1^Heart Center, Al Jalila Children’s Hospital, Al Jaddaf, Dubai, United Arab Emirates; ^2^Department of Pediatrics and Adolescent Medicine, American University of Beirut Medical Center, Beirut, Lebanon

**Keywords:** Isolated innominate artery, Isolated left brachiocephalic artery, DiGeorge syndrome, Right aortic arch

## Abstract

Isolated left innominate artery with right-sided aortic arch and bilateral patent ducts is a rare congenital abnormality. It has been described in association with various intracardiac lesions. In this report we describe the clinical presentation, investigations, and surgical management of a very peculiar case of a 4-year-old female with isolated left brachiocephalic trunk and bilateral patent arterial ducts without associated congenital heart defect, in the setting of 22q11 microdeletion.

## Introduction

 Congenital anomalies involving the aortic arch are estimated to complicate 15%–20% of congenital cardiac diseases.^1^ A rare congenital anomaly involving the innominate artery receiving blood supply form the pulmonary artery via a patent ductus has been scarcely reported in the literature. Such malformation is usually coupled to right sided aortic arch and septal defects.^2^ It has been also reported in association with variety of congenital cardiac defects and syndromes including septal defects, aortic coarctation, aortic atresia, transposition of the great vessels, Down syndrome, DiGeorge syndrome, and the constellation of features encompassed by Coloboma, Heart defects, Atresia of the choanae, Retardation of growth and development, Genital anomalies, and Ear anomalies (CHARGE)^3-6^

 The clinical presentation is variable and is primarily influenced by accompanying congenital anomalies or syndromes. Patients may exhibit no symptoms or present with pulmonary arterial hypertension, cyanosis, dyspnea or even congestive heart failure.^2,7^ Others might only exhibit upper extremity muscle weakness.^7^ In this manuscript, we report a rare case of a 4-year-old female diagnosed with DiGeorge syndrome, who has an isolated left innominate artery, a right aortic arch and bilateral patent arterial ductus, without associated intra-cardiac defects.

## Case Report

 A 4-year-old female was referred to our center for the management of residual patent ductus arteriosus (PDA). She was chronically followed up by her pediatrician for persistent hypocalcemia and seizure disorder and was maintained on calcium and vitamin D supplementation. At the age of one year, she underwent PDA closure using patent ductus Occluder type I device at another institution. On physical examination, the patient was active and noncyanotic. Growth parameters revealed below-average weight (10th percentile) and height within the low-normal range (22nd percentile). No syndromic features or other apparent anomalies were observed. On auscultation, heart sounds were normal except for a grade 3/6 continuous murmur heard best over the upper chest and back. The examination also revealed a diminished left radial pulse in comparison to the right, while femoral pulses were palpable and equal bilaterally. Furthermore, there was a striking difference in blood pressure between her upper extremities, evidenced by a systolic blood pressure reading of 50 mmHg in the left arm, as compared to 100 mm Hg in the right arm. Electrocardiogram showed a normal sinus rhythm with increased voltage suggestive of left ventricular hypertrophy. The two-dimensional echocardiogram showed mild dilation of the left ventricle with normal function. There was a right aortic arch with a device seen closing a right PDA. A high-velocity continuous flow was detected in the left pulmonary artery (LPA) that was attributed to a residual shunt across the PDA device. Fluorescent in situ hybridization (FISH) deletion study in addition to flow cytometry were performed which revealed microdeletion of chromosome 22q11.2, consistent with DiGeorge syndrome.

 Cardiac catheterization was performed with the intention to close a residual shunt across the aforementioned PDA device. The angiography showed the right aortic arch and the PDA device closing a right sided PDA without residual shunt. A solitary left-sided innominate artery was seen originating directly from the main pulmonary artery (MPA) through a large left PDA. It filled in a retrograde manner from the left-sided vertebral artery via the cerebral circulation (Figure 1). The pulmonary artery was dilated, and the mean PA pressure was 26 mm Hg. The systolic pressure in the left brachiocephalic artery was 50 mm Hg compared with an ascending aortic systolic pressure of 100 mm Hg.

**Figure 1:**
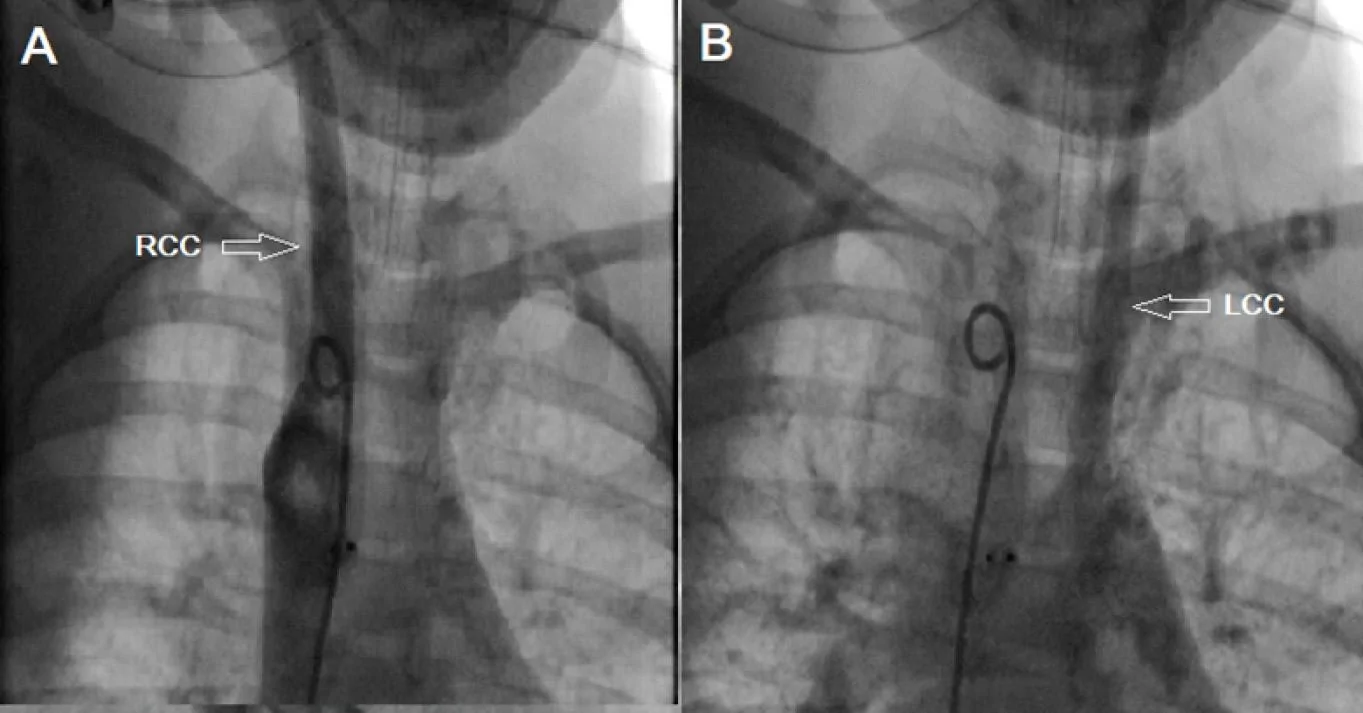
Selective angiogram in the right common carotid (RCC) via a right aortic arch in an antero-posterior projection A: early phase; B: late phase showing the left common carotid artery, filled via the left vertebral artery and the cerebral circulation

 Computed tomography angiography was performed to further delineate the complex aortic arch and ductal anatomy, confirm the origin and course of the left brachiocephalic artery, and exclude the presence of a complete vascular ring, which was essential for surgical planning (Figure 2).

**Figure 2:**
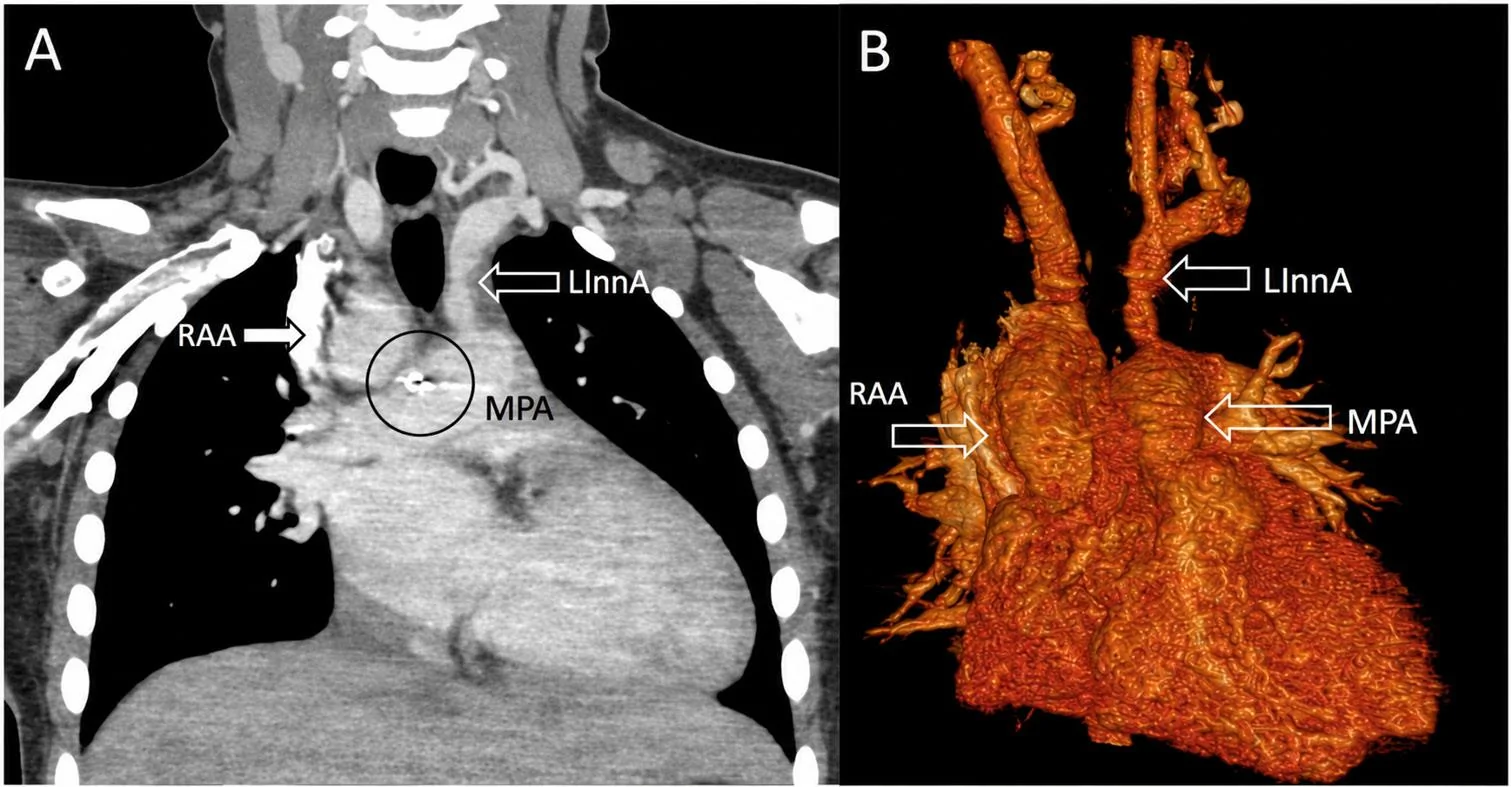
Computed Tomography angiogram of the chest showed A: right aortic arch (RAA black arrow), the ductus arteriosus device (black circle) and the origin of the left innominate artery (LinnA) from the main pulmonary artery (MPA). B: a three-dimensional rendered image from the same CT angiogram showing the same findings. Please note the absence of a complete vascular ring

 Surgical correction was undertaken to eliminate the left-to-right shunt and restore adequate systemic perfusion to the left upper extremity and cerebral circulation. The procedure was performed via a median sternotomy without the use of cardiopulmonary bypass, as intracardiac repair was not required.

 After systemic heparinization, the MPA, LPA, left ductal structure, and the right-sided aortic arch were carefully exposed and mobilized. Intraoperative inspection confirmed a large left-sided ductus arteriosus arising from the MPA, which progressively dilated to form a brachiocephalic trunk measuring approximately 20 mm in diameter, giving rise to both the left common carotid and left subclavian arteries. Due to the long distance between this common trunk and the right-sided aortic arch, direct end-to-end anastomosis was deemed unfeasible.

 To achieve anatomical reconstruction, a segment of the proximal LPA was harvested and fashioned as an interposition graft. The proximal end of this graft was anastomosed end-to-side to the left aspect of the right-sided aortic arch using continuous polypropylene sutures. Subsequently, the anomalous brachiocephalic trunk was ligated and transected from its origin at the MPA and anastomosed end-to-end to the distal end of the interposition graft, thereby re-establishing antegrade systemic flow to the left carotid and subclavian arteries.

 Following completion of the vascular reconstruction, the LPA was mobilized from the hilum and re-anastomosed to the MPA to preserve pulmonary arterial continuity and avoid LPA stenosis. Hemostasis was achieved, and intraoperative assessment confirmed adequate pulsatile flow through the reconstructed brachiocephalic artery and balanced pulmonary artery flow.

 Two weeks following the operation, the patient was asymptomatic with palpable and equal radial pulses. Follow up echocardiography at two weeks showed normal LV size and systolic function, with resolution of pulmonary arterial hypertension without residual shunting at the arterial levels. Antegrade pulsatile flow was seen in the left carotid and proximal subclavian artery.

 The patient is planned to continue regular follow-up in the pediatric cardiology outpatient clinic with serial echocardiography according to our institutional protocol, including assessment at 1 month following surgery, then at 6 months, and annually thereafter if no abnormalities are identified. If any concerns arise, computed tomography angiography (CTA) or cardiac catheterization will be performed for further evaluation.

## Discussion

 The events leading to the development of the aortic arch system have yet to be fully elucidated. However, it is suggested to include a series of steps through which six pairs of vessels, that connect the trunco-aortic sac with the paired dorsal aorta, either regress or persist. Ultimately, they merge to create the final descending aorta.^8^ It is proposed that migration of neural crest cells into the pharyngeal arches plays a significant role in this process.^9^ In our patient, as in other anomalies, the lesion could be theoretically explained by the “Edwards double arch hypothetical model”: Regression of the left ventral aorta and the left fourth arch distal to the seventh intersegmental artery resulting in isolated left innominate artery that is linked to the MPA via a left-sided patent ductus. In this patient the concomitant patency of the right PDA is very peculiar. Figure 3 illustrates the proposed embryologic mechanism underlying the observed anatomy, using Edwards’ double aortic arch hypothetical model to depict the sequential stages of vascular remodeling that could explain the development of an isolated left innominate artery in the setting of a right-sided aortic arch. Furthermore, the association of various arch anomalies with chromosome 22q11 microdeletion suggests a genetically-influenced process implicated in the development of arch abnormalities.^10^ Indeed, DiGeorge patients might have cono-truncal anomalies, and interestingly, more than half of those have associated arch abnormalities.^11^ Additionally, approximately 25% of patients who exhibit isolated arch anomalies without defects within the heart have a 22q11 deletion.^12^

**Figure 3:**
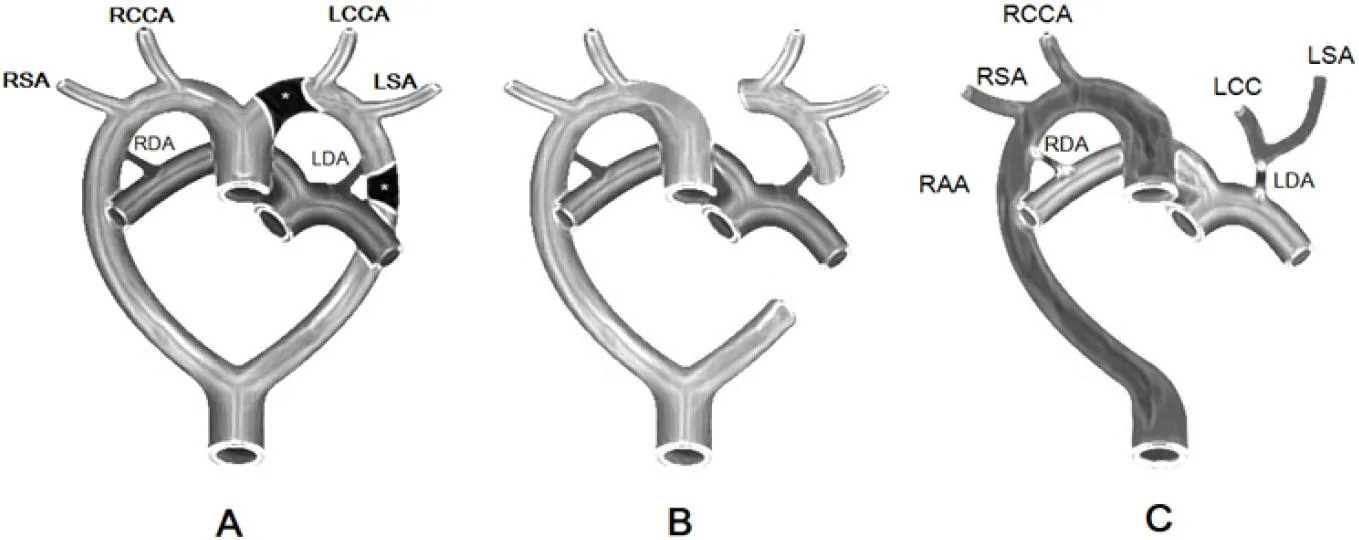
Schematic representation of the findings in the case report. A: based on Edwards double arch hypothetical model, the presumed areas of dissolution are shown (white stars). B: intermediate phase of the development of the isolated left innominate artery. C: final representation of the findings shown in figures 1 and 2. LCCA: left common carotid artery. LDA left ductus arteriosus. LSA: left subclavian artery. RAA: right aortic arch. RCCA: right common carotid artery. RDA: right ductus arteriosus. RSA: right subclavian artery

 A right-sided aortic arch coupled to an isolated left-sided arch vessel is relatively uncommon. Three different forms have been noted, with the isolated left subclavian being the most common. Furthermore, most cases (98%) of right arch with a PDA are associated with congenital cardiac defects.^13,14^ Rad and Pouraliakbar identified 31 cases of isolated brachiocephalic and right aortic arch through their literature review spanning from 1966 to 2018, with only seven patients devoid of associated congenital cardiac defect.^15^ To our knowledge, only three cases were reported with a similar arch anomaly, bilateral PDA and normal intracardiac anatomy.^16-18^

 Patients who display symptoms secondary to their anomalies are usually referred for surgical management. The most preferred intervention is to re-attach the left innominate vessel to the aortic arch.^2,19^ In our patient, we decided to manage surgically to restore the systemic perfusion and eliminate left-to-right shunting. Moreover, re-establishing the continuity between the aortic arch and brachiocephalic artery using autologous tissue is theoretically advantageous. We did not see any significant postoperative stenosis either at the takeoff from the arch nor at the insertion site into the brachiocephalic trunk, however, long term follow up is needed.

 This anomaly should be suspected in patients showing upper extremity pulse or blood pressure discrepancies or unexplained continuous pulmonary artery flow on echocardiography. Cross-sectional imaging, preferably CT angiography or cardiac MRI, is essential to confirm the diagnosis and delineate the vascular anatomy. Cardiac catheterization may provide useful hemodynamic data when surgical intervention is considered. From a management perspective, surgical re-attachment of the isolated vessel to the aortic arch is recommended when there is evidence of systemic hypoperfusion or significant shunting. When direct anastomosis is not feasible, using autologous tissue such as a segment of the left pulmonary artery remains a safe and durable alternative.

## Conclusion

 Isolated brachiocephalic trunk in the presence of bilateral PDAs and normal intracardiac anatomy is a rare anomaly. Clinically, it should be suspected whenever there is disparity in pulse or blood pressure measurement between the upper extremities. Surgical repair utilizing autologous LPA to establish continuity between the aortic arch and brachiocephalic artery could be used when implantation is not feasible.

## Competing Interests

 The authors have no relevant financial or non-financial interests to disclose.

## Ethical Approval

 This study was performed in line with the principles of the Declaration of Helsinki. 
